# Vitamin D deficiency as a risk factor for the development of autoantibodies in patients with ASIA and silicone breast implants: a cohort study and review of the literature

**DOI:** 10.1007/s10067-017-3589-6

**Published:** 2017-03-17

**Authors:** Maartje J. L. Colaris, Rene R. van der Hulst, Jan Willem Cohen Tervaert

**Affiliations:** 1 Faculty of Health, Medicine and Life Sciences, Maastricht University, Universiteitssingel 40 6229 ER, Maastricht, the Netherlands; 2 Department of Reconstructive, Plastic and Hand Surgery, Maastricht University Medical Center, Maastricht, the Netherlands; 3 Clinical and Experimental Immunology, Reinaert Clinic, Maastricht, the Netherlands

**Keywords:** Autoantibodies, Autoimmune diseases, Vitamin D, Vitamin D deficiency, Vitamin D supplementation

## Abstract

The development of autoimmunity and/or autoimmune diseases is multifactorial. Vitamin D is one of the factors that might play a role. We postulated that both the presence of adjuvants and insufficient levels of vitamin D may result in the development of autoimmunity in patients with autoimmune/inflammatory syndrome induced by adjuvants (ASIA) in relation to silicone implant incompatibility. We measured vitamin D levels in 135 patients with ASIA in relation to silicone implant incompatibility and related findings to the presence of autoantibodies that are commonly used to diagnose systemic autoimmune diseases. Furthermore, we systematically reviewed the literature regarding vitamin D deficiency as a risk factor for the development of autoantibodies. Vitamin D measurements were available for analysis in 131 of 135 patients with ASIA in relation to SIIS. Twenty-three patients (18%) tested positive for autoantibodies, from which 18 patients (78%) had either a vitamin D deficiency or insufficiency (median vitamin D level 60.5 mmol/L), whereas five patients (22%) had sufficient vitamin D levels. The risk to develop autoantibodies was significantly increased in vitamin D deficient and/or insufficient patients [RR 3.14; 95% CI, 1.24–7.95; *p* = 0.009]. Reviewed literature suggested an association between vitamin D levels and the presence and/or titer levels of autoantibodies in different autoimmune diseases. From our current study and from our review of the literature, we conclude that vitamin D deficiency is related to the presence of autoantibodies. Whether vitamin D supplementation results in a decrease of autoimmunity needs to be studied prospectively.

## Introduction

Silicone breast implants may be associated with nonspecific symptoms such as fatigue, myalgia, arthralgia, pyrexia, dry mouth, dry eyes, and cognitive impairment [1–3]. Explantation of the breast implants results in improvement of the symptoms in 50–80% of the patients [1, 4]. At present, it is still controversial whether silicone-filled breast implants increase the risk of autoimmunity [3, 5, 6]. Recently, however, it has been suggested that an increased prevalence of autoimmune diseases such as rheumatoid arthritis and Sjögren’s syndrome exists in these patients [6]. Moreover, immune deficiency may occur in these patients at an increased frequency [3]. Finally, several autoantibodies have been reported to occur in silicone breast implant patients at an increased frequency [7–11]. Also, these findings are, however, controversial [11, 12].

Vitamin D is known to be a crucial factor in the calcium homeostasis. Vitamin D, however, is also essential for immunity. Importantly, vitamin D deficiency may represent a global health problem that has been underestimated for many years [13]. Over the last decades, it has been shown that vitamin D deficiency is associated with an increased risk for infectious diseases [14]. Moreover, a number of autoimmune diseases, including multiple sclerosis, type I diabetes, inflammatory bowel disease, systemic lupus erythematosus, and rheumatoid arthritis, are reported to be associated with vitamin D deficiency [15–17].

Several mechanisms by which vitamin D acts as a regulatory agent for the innate as well as the adaptive immune system have been described [18]. Firstly, cells of the immune system have been shown to be direct targets of vitamin D metabolites. Secondly, many immune cells contain enzymes of the cytochrome P (CYP) family and are thus able to convert 25(OH)D into calcitriol, the biologically active form of vitamin D [18, 19].

Since vitamin D is known to be a potent regulator of the immune system [18] and vitamin D deficiency is hypothesized to contribute to B cell hyperactivity [19], we postulate that vitamin D deficiency is a risk factor for the development of autoantibodies in patients with autoimmune/inflammatory syndrome induced by adjuvants (ASIA) in relation to silicone implant incompatibility syndrome (SIIS) [3, 20]. To study this, we examined vitamin D levels in patients with ASIA in relation to SIIS and related findings to the presence of autoantibodies. Furthermore, we reviewed the effect of vitamin D deficiency as a risk factor for the development and/or production of autoantibodies.

## Patients and methods

### Study population

One hundred thirty-five consecutive ASIA in relation to SIIS patients were included in the current study. The patients were prospectively evaluated between January 2014 and September 2015 by one of us (JWCT). A diagnosis of ASIA was made based on criteria as previously described [2]. In short, patients were diagnosed as suffering from ASIA when either two major or one major and two minor criteria were present. Criteria are summarized in Table 1.

**Table 1:** Criteria for the diagnosis of ASIA

Major criteria
• Exposure to an external stimulus (infection, vaccine, silicone, adjuvant) prior to clinical manifestations. • The appearance of “typical” clinical manifestations: Myalgia, myositis, or muscle weakness Arthralgia and/or arthritis Chronic fatigue, un-refreshing sleep, or sleep disturbances Neurological manifestations (especially associated with demyelination) Cognitive impairment, memory loss Pyrexia, dry mouth • Removal of inciting agent induces improvement. • Typical biopsy of involved organs
Minor criteria:
The appearance of autoantibodies or antibodies directed at the suspected adjuvant • Other clinical manifestations (i.e., irritable bowel syndrome) • Specific HLA (i.e., HLA DRB1, HLA DQB1) • Evolvement of an autoimmune disease (i.e., multiple sclerosis, systemic sclerosis)

*ASIA* autoimmune/inflammatory syndrome induced by adjuvants

### Laboratory investigation

All patients underwent laboratory evaluations including measurements of vitamin D, antinuclear antibodies (ANA), antibodies to extractable nuclear antigens (ENA), anti-cardiolipin antibodies (anti-CL), anti-β2 glycoprotein-1 antibodies, anti-neutrophil cytoplasmic antibodies (ANCA), IgM rheumatoid factor (RF), and anti-cyclic citrullinated peptide (anti-CCP) antibodies [21–27]. If a positive ANA was found, serum was tested for anti-double-stranded DNA antibodies (anti-dsDNA) [26], and if the ANCA test was positive, samples were additionally tested for PR3-ANCA and MPO-ANCA [25].

Vitamin D status was evaluated by measurement of serum 25(OH)D levels with a chemiluminescent immunoassay method by Roche Cobas, Roche, Basel, Switzerland. Serum 25(OH)D levels below 50 nmol/L (20 ng/mL) were considered as vitamin D deficiency. Vitamin D insufficiency was defined as ≥50 and <75 nmol/L (21–29 ng/mL), whereas vitamin D sufficiency was defined as ≥75 nmol/L (30 ng/mL) [28, 29].

### Statistical analysis

For statistical analysis of the results, a chi-square test with a 0.05 two-sided significance level was used (SPSS 22.0 software, IBM Corp, Armonk, NY).

## Review

### Type of studies and outcome measures

All types of studies comparing vitamin D status and autoantibodies in patients with or without autoimmune diseases were included in this review. Meta-analysis and systematic review articles were excluded. Only articles published in English were used.

A full paper review was performed when abstracts described vitamin D levels in relation to the presence of autoantibodies and/or antibody titers. Exclusion of articles was based on the absence of (statistical) analysis of the (possible) association between serum vitamin D levels and the presence of autoantibodies and/or antibody titers.

### Search methods

A search in MEDLINE, PubMed, and the Cochrane database from their inception to February 2016 was performed. The used search terms in the PubMed NLM catalog Medical Subject Heading database relevant to this review were “Vitamin D,” “Vitamin D [Mesh],” “Vitamin D deficiency [Mesh],” “Autoantibodies,” and “Autoantibodies [Mesh].” Abstracts from 176 search hits were screened for eligibility.

### Study selection

**Fig. 1:**
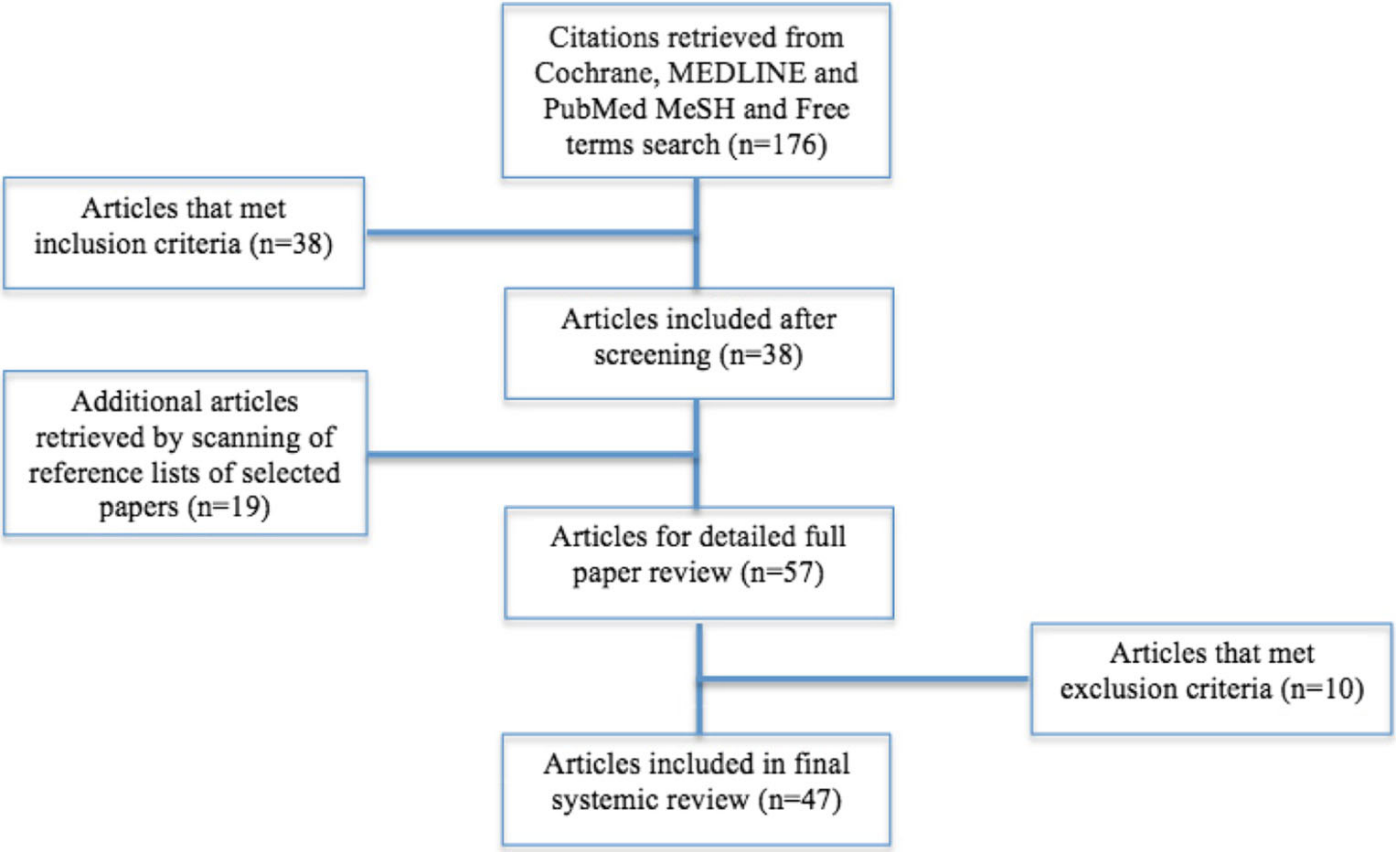
Flow diagram of study enrolment in this review

Titles and abstracts were screened for eligibility according to the inclusion criteria by the first author. When included outcome measures were unclear after regarding the abstract, articles were retrieved in full text for additional assessment. After screening the 176 PubMed abstracts according to the inclusion criteria, 38 articles were retrieved for full paper review. Also, a manual search of the reference lists of the selected articles was performed, which resulted in 19 additional articles for the full paper review. Only published data were used. A total of 57 articles were retrieved for detailed full paper review. Criteria for exclusion were met in 10 articles, leaving 47 articles that were included in the final systematic review. A flow diagram of the selection process is shown in Fig. 1.

### Data extraction

Data relevant to the pre-stated outcome measures, the characteristics of the study, participants, and relevant statistical analysis of a (possible) association between the outcome measures were abstracted for this review.

## Results

Vitamin D measurements were available for analysis in 131 of 135 patients with ASIA in relation to SIIS (Table 2). Thirty-three patients (25%) presented with vitamin D deficiency (<50 nmol/L), 37 (28%) with vitamin D insufficiency (*≥*50 and <75 nmol/L), and 61 (47%) with vitamin D sufficiency (*≥*75 nmol/L). No differences were observed in clinical characteristics between groups.

**Table 2:** The presence of clinical manifestations in vitamin D subgroups

Symptom		No. of patients	
	Vitamin D deficiency (<50 nmol/L) *n* = 33	Vitamin D insufficiency (≥50–75 nmol/L) *n* = 37	Vitamin D sufficiency (≥75 nmol/L) *n* = 61
Myalgia, myositis, or muscle weakness	18	17	41
Arthralgia and/or arthritis	31	33	58
Fatigue, unrefreshing sleep or sleep disturbances	32	37	60
Neurological manifestations^a^	7	7	16
Cognitive impairment, memory loss	25	30	47
Pyrexia	23	22	48
Dry eyes and/or dry mouth (sicca)	27	24	47

^a^Neurological manifestations: TIA/CVA or demyelination

Twenty-three patients (18%) tested positive for autoantibodies. In these 23 patients, one or more antibodies were detected (Table 3). Eighteen of these patients (78%) had either a vitamin D deficiency or insufficiency (median vitamin D level 60.5 mmol/L), whereas five patients (22%) had sufficient vitamin D levels. The risk to develop autoantibodies was significantly increased in vitamin D deficient and/or insufficient patients [RR 3.14; 95% CI, 1.24–7.95; *p* = 0.009].

**Table 3:** Autoantibodies detected in 23^b^ patients with ASIA in relation to SIIS

Autoantibodies	No. of patients
ANA/ENA^a^ Anti-CL ANCA IgM-RF Anti-CCP	6 7 4 4 3

*ANA* antinuclear antibodies, *anti-dsDNA* anti-double-stranded DNA antibodies, *anti-SSA* anti-Sjögren’s syndrome-related antigen A, *anti-SSB* anti-Sjögren’s syndrome-related antigen B, *anti-CENPB* anti-centromere protein B, *anti-Scl-70* anti-topoisomerase I, *anti-CL* anti-cardiolipin, *ANCA* anti-neutrophil cytoplasmic antibodies, *IgM-RF* IgM rheumatoid factor, *anti-CCP* anti-cyclic citrullinated peptide antibody

^a^ANA specificities: anti-dsDNA (*n* = 1), anti-SSA (*n* = 2), anti-SSB (*n* = 1), anti-CENP-B (*n* = 1), anti-Scl70 (n = 1)

^b^One patient had two autoantibodies (anti-CCP and ANCA)

## Review

Forty-seven studies were reviewed (17 case–control studies, 19 cross-sectional studies, 1 cross-sectional case–control study, 10 cohort studies). The selected articles will be presented according to five subcategories: autoimmune thyroid diseases, connective tissue diseases, inflammatory arthritides, multiple sclerosis, and other studies.

### Autoimmune thyroid diseases

In several epidemiological studies, the association between vitamin D levels and the presence of autoimmune thyroid diseases (AITDs) has been investigated [30]. Kivity et al. examined serum vitamin D levels in relation to anti-thyroid peroxidase (anti-TPO) and anti-thyroglobulin (anti-TG) antibodies in 92 patients with thyroid diseases (28 patients with Hashimoto’s thyroiditis, 22 patients with Graves’ disease, 42 patients without evidence for autoimmunity) and 98 age-matched healthy control subjects [31]. They documented a significantly higher prevalence of vitamin D deficiency (<10 ng/mL) in patients with thyroid diseases (58/92) compared to healthy controls (30/98) (*p* < 0.001). Vitamin D deficiency was present particularly in patients with Hashimoto’s thyroiditis (22/28; *p* = 0.001) and in patients with Graves’ disease (14/22; *p* = 0.01). As a consequence, vitamin D deficiency was found to be correlated with the presence of anti-thyroid antibodies (*p* = 0.01) [31]. Choi et al. observed in a cross-sectional study that serum 25(OH)D levels were significantly lower in anti-TPO-positive (427/2793) compared to anti-TPO-negative (2366/2793) female euthyroid subjects (*p* = 0.03) [32]. In a subgroup analysis of pre- (*n* = 908) and postmenopausal (*n* = 1885) women, the serum 25(OH)D levels were significantly lower in pre-menopausal women with anti-TPO antibodies (*p* = 0.049) but not in postmenopausal women with these antibodies (*p* = 0.186) [32]. Also, Goswami et al. found a significant inverse correlation between serum vitamin D levels and anti-TPO positivity (*p* = 0.04) in an Asian Indian population [33]. Arslan et al. found anti-TPO positivity more frequently observed in healthy volunteers with severe (<10 ng/mL) and moderate (10–20 ng/mL) vitamin D levels, compared to those with normal vitamin D levels (≥20 ng/mL) [34]. Additionally, in antibody-positive patients, a significant inverse correlation between anti-TPO and anti-TG titers and 25(OH)D levels (*p* = 0.017 for anti-TPO and *p* = 0.05 for anti-TG) was found [34]. In a case–control study, Unal et al. found that anti-TG and anti-TPO titers were significantly higher in vitamin D-deficient AITD patients (183/281) than in vitamin D-sufficient AITD patients (98/281) (*p* = 0.02 and *p* = 0.003, respectively, for anti-TG and anti-TPO) [35]. In this study, the 281 AITD patients consist of 254 patients with Hashimoto thyroiditis and 27 patients with Graves’ disease. Several other studies also found a negative correlation between serum vitamin D levels and anti-thyroid antibody levels [36–40]. Mazokopakis et al. found that anti-TPO levels were significantly higher in vitamin D-deficient patients with Hashimoto’s thyroiditis (HT) (*n* = 186) compared to HT patients with no vitamin D deficiency (*n* = 32) (364 ± 181 vs. 115.8 ± 37.1 IU/mL, *p* < 0.0001) [36], whereas Bozkurt et al. found that serum 25(OH)D levels were inversely correlated with anti-TPO (*p* < 0.001) and anti-TG levels (*p* < 0.001) in subjects with HT [36]. Furthermore, Bozkurt observed that serum 25(OH)D levels in HT patients were significantly lower than in healthy controls [37]. Shin et al. found that AITD patients (65 Hashimoto’s thyroiditis and 46 Graves’ disease) with anti-thyroid antibodies had lower levels of serum 25(OH)D than control patients without positive anti-thyroid antibodies (12.6 ± 5.5 vs. 14.5 ± 7.3 ng/mL, respectively, *p* < 0.001) [38]. They also found that 25(OH)D levels were inversely correlated with the anti-TPO levels (*p* < 0.001) in the HT and Graves’ patients. More recently, Wang et al. analyzed a total of 1714 Chinese healthy adults and reported that subjects that tested positive for anti-TG (*n* = 315) had lower serum 25(OH)D levels compared to subjects who were anti-TG negative (*p* < 0.01) [39]. Furthermore, they observed that higher anti-TG titers were associated with lower 25(OH)D levels independent of age, ethnicity, and TSH levels in females but not in males (*p* = 0.014). Finally, in non-lactating women with postpartum thyroiditis, anti-TPO and anti-TG titers were found to be inversely correlated with vitamin D levels (*p* < 0.001) [40].

In contrast, D’Aurizio et al. did not observe statistically significant differences in vitamin D levels between healthy controls and either patients with HT and/or Graves’ disease (*n* = 100) and anti-TPO and/or anti-TSH receptor autoantibody positivity [41]. Also, Ma et al. did not find serum 25(OH)D levels to be associated with anti-TPO or anti-TG levels in 70 patients with newly diagnosed HT [42]. However, an association between serum 25(OH)D levels and anti-TSH receptor autoantibody levels was observed in their 70 patients (*p* = 0.036) [42]. Yasuda et al. evaluated 26 female patients with newly onset Graves’ disease and found no association between serum 25(OH)D levels and anti-TSH receptor autoantibody levels [43]. In women with polycystic ovary syndrome and positive anti-TPO and/or anti-TG antibody levels, no correlation between 25(OH)D levels and these antibodies was seen [44]. Finally, Effraimidis et al. reported a longitudinal study with a 5-year follow-up period in which serum 25(OH)D levels of 521 euthyroid subjects were analyzed and related to the occurrence of thyroid peroxidase antibodies (anti-TPO) [45]. Subjects were euthyroid women who had first- or second-degree relatives with overt AITD who had normal TSH and no thyroid antibodies at baseline. Subjects that developed anti-TPO within a period of 5 years were compared to subjects who remained anti-TPO negative, matched for age, BMI, smoking status, estrogen use, month of blood sampling, and duration of follow-up [45]. Sixty-seven subjects became positive for anti-TPO during follow-up, without developing abnormal TSH. No association with serum 25(OH)D levels was reported.

In conclusion, several studies suggested an association between the presence of anti-TPO and/or anti-TG and vitamin D levels and/or between autoantibody level and vitamin D level (Table 4). In a prospective study, however, vitamin D levels were not found to be a risk factor for the development of anti-TPO.

**Table 4:** Studies in which vitamin D levels are correlated to thyroid autoantibodies in thyroid autoimmune diseases (AITDs)

Author	Year	Country	Subjects	Vitamin D (ng/ml)	Thyroid autoantibodies	*P* value^*^	Reference
Kivity S. et al.	2011	Hungary	92 thyroid diseases^a^ (28 HT, 22 GD, 42 negative Ab), 98 CTRL	NR	anti-TPO, anti-TG	0.01	[31]
Choi Y.M. et al.	2014	Korea	427 TPOAb +, 2366 TPOAb – (CTRL)	22.0 ± 0.6 vs. 23.5 ± 0.3	anti-TPO	0.03	[32]
Goswami et al.	2009	India	642 (559 vit. D ≤ 25 nmol/l, 83 vit. D > 25.0 nmol/l)	14.5 ± 3.5 vs. 37.5 ± 16.3	anti-TPO	0.04^†^	[33]
Arslan M.S. et al.	2015	Turkey	155 volunteers (53 group 1^f^, 61 group 2^g^, 41 group 3^h^)	<10 vs. 10–19.9 vs. ≥ 20	anti-TPO, anti-TG	0.017 0.05	[34]
Unal A.D. et al.	2014	Turkey	281 AITDs (254 HT, 27 GD), 124 CTRL^d^	13.4 ± 3.6 vs. 29.5 ± 10	anti-TPO, anti-TG	0.003 0.02	[35]
Mazokopakis et al.	2015	Greece	218 HT (186 vit. D deficiency, 32 vit. D sufficiency)	14.6 ± 7.2	anti-TPO, anti-TG	<0.00001 NS	[36]
Bozkurt N. et al.	2013	Turkey	360 AITDs (180 HT, 180 HT^i^), 180 CTRL	11.4 ± 5.2 vs. 13.1 ± 5.9 vs. 5.4 ± 6.8	anti-TPO, anti-TG	<0.001 <0.001	[37]
Shin D.Y. et al.	2014	Korea	111 AITDs, 193 non-AITDs	12.6 ± 5.5 vs. 14.5 ± 7.3	anti-TPO	<0.001	[38]
Wang X. et al.	2015	China	1714 Chinese adults (1197 females, 517 males)	13.61 (10.05-18.47)	anti-TG anti-TG titers	<0.01 0.014	[39]
Krysiak R. et al.	2016	Poland	38 PPT^k^, 21 CTRL^l^	25.0 ± 10 vs. 40.0 ± 12	anti-TPO, anti-TG	<0.001 <0.001	[40]
D’Aurizio F. et al.	2015	Italy	100 AITDs (52 HT, 48 GD), 126 CTRL	25.8 vs. 30	anti-TPO	NS	[41]
Ma J. et al.	2015	China	140 AITDs (70 HT, 70 GD), 70 CTRL^e^	31.00-31.71 vs. 1.33	anti-TPO, anti-TG	NS	[42]
Yasuda T. et al.	2012	Japan	26 GD, 46 CTRL^j^	14.4 ± 4.9 17.1 ± 4.1	anti-TPO, anti-TG	NR	[43]
Muscogiuri G. et al.	2015	Italy	50 PCOS (12 TPOAb/TGAb +), 38 TPOAb/TGAb -)	32.0 ± 22.6, 49.6 ± 19.9	anti-TPO, anti-TG	0.21 0.16	[44]
Effraimidis G. et al.	2012	Netherlands	67 AITDs^b^, 67 CTRL^c^	21.6 ± 9.2 vs. 21.2 ± 9.3	anti-TPO	NS	[45]

AITDs = Autoimmune Thyroid Diseases; HT = Hashimoto's thyroiditis; GD = Graves' disease; CTRL = Controls; NR = Not reported; anti-TPO = Antithyroid peroxidase; anti-TG = anti-thyroglobulin antibodies; PCOS = Polycystic Ovary Syndrome.

^*^Correlation between vitamin D levels and positive thyroid antibodies

^†^Controlled for age

^a^58 vitamin D deficiency (<10 ng/ml), 34 vitamin D >10 ng/ml

^b^anti-TPO negative at baseline (cases)

^c^anti-TPO negative (controls)

^d^age matched healthy controls

^e^matched for sex, age, and smoking status with the GD patients

^f^Vitamin D level < 10 ng/ml

^g^Vitamin D level 10–19.9 ng/ml

^h^Vitamin D level ≥ 20 ng/ml

^i^sex-, age-, and body mass index (BMI)-matched euthyroid subjects with newly diagnosed HT

^j^healthy female subjects

^k^non-lacting L-thyroxine-treated women with postpartum thyroiditis (PPT)

^l^matched healthy postpartum women

### Connective tissue diseases

Low levels of vitamin D are often found in systemic lupus erythematosus (SLE) patients [46, 47]. Vitamin D levels are lower in postmenopausal SLE patients (*n* = 80) compared to pre-menopausal SLE patients (*n* = 80) (*p* = 0.02) [48]. No association, however, could be observed between vitamin D food intake and supplements and risk of the development of SLE [49]. ANA-positive SLE patients are more likely to have vitamin D deficiency (≤20 ng/mL) compared to ANA-negative SLE controls (*p* = 0.011) [46]. Also, anti-dsDNA autoantibody levels increased from normal vitamin D concentrations, to insufficient vitamin D levels and from insufficient to vitamin D-deficient patient subsets as found in 177 Hungarian SLE patients (*p* = 0.021) [48]. In women with newly diagnosed SLE, Bonakdar et al. also found higher titers of anti-dsDNA antibodies with more severe vitamin D deficiency (*p* < 0.001) [50]. Also, Mok et al. showed a significant negative correlation between 25(OH)D levels and anti-dsDNA levels (*p* = 0.02) or anti-C1q levels (*p* = 0.02) in their SLE patients (*n* = 290) [51]. In contrast, Thudi et al. found higher levels of anti-dsDNA in a small group of female lupus patients with levels of vitamin D that were greater than 47.7 nmol/L compared to subjects with 25(OH)D levels lower than 47.7 nmol/L (*p* = 0.0069) (*n* = 37) [52]. Finally, in a small study performed in 28 Saudi children with SLE, levels of 25(OH)D tended to be inversely correlated with anti-dsDNA titers and ANA [53].

Also, in other connective tissue diseases, low levels of vitamin D are found to be correlated with autoantibodies. In patients with Sjögren’s syndrome, an inverse correlation between the concentrations of 25(OH)D and the titers of IgM rheumatoid factor were found (*n* = 25) [54]. In patients with mixed connective tissue disease (MCTD), higher serum levels of anti-U1-RNP (anti-ribonucleoprotein) antibodies were found in patients with low vitamin D levels (*p* = 0.022) [55]. Also, in 161 undifferentiated connective tissue disease (UCTD) patients, autoantibody profiles showed that serum levels of anti-U1-RNP, anti-SSA, and anti-CCP antibodies were inversely correlated to vitamin D levels (*p* < 0.05) [56]. During an average follow-up period of 2.3 years, 35/161 patients developed an established connective tissue disease. Vitamin D levels were lower in these patients compared to the vitamin D levels as found in the 126/161 UCTD patients that did not develop an established CTD (14.7 ± 6.45 vs. 33.0 ± 13.4 ng/mL, *p* = 0.0001) [56]. In a cross-sectional study in 23 pre-menopausal women with primary anti-phospholipid syndrome (APS), serum vitamin D levels and IgG and IgM anti-cardiolipin (anti-CL) antibodies were investigated and compared to vitamin D levels in 23 age- and race-matched healthy controls [57]. Lower serum levels of 25(OH)D [21.64 mg/dL (11.26) vs. 28.59 mg/dL (10.67), *p* = 0.039] were found in the APS patients when compared to controls. No correlation, however, was found between vitamin D levels and IgG anti-CL levels (*p =* 0.222) and/or IgM anti-CL levels (*p =* 0.535). In a large multinational study in patients with systemic sclerosis (SSc), vitamin D concentrations and the presence of anti-nuclear antibodies (ANA), anti-SCL70 antibodies, and RF were determined [58]. Three hundred twenty-seven serum samples of European patients with SSc and 141 samples of healthy controls were studied. Patients with SSc had significantly lower serum vitamin D concentrations compared to healthy controls (*p* < 0.001). An inverse relation between RF positivity and the vitamin D concentration was found (*p* < 0.001). No association was found between vitamin D levels and the presence of ANA and/or anti-SCL70.

In conclusion, an inverse correlation between vitamin D levels and the presence of autoantibodies in connective tissue diseases has been described (Table 5). In several studies, vitamin D deficiency was found to be inversely correlated with autoantibody titers in connective tissue diseases, although findings are at present not conclusive.

**Table 5:** Studies in which vitamin D levels are correlated to CTD autoantibodies in connective tissue diseases (CTDs)

Author	Year	Country	Subjects	Vitamin D (ng/ml)	Positive CTD autoantibodies	*P* value^h^	Reference
Ritterhouse L. et al.	2011	USA	32 SLE, 32 CTRL^a^	17.3 vs. 17.4^*^*^ vs. 29.4^^*^*^	ANA	<0.01	[46]
Szodoray P. et al.	2011	Hungary	177 SLE (160 females, 17 males)	<15 vs. 15–30 vs. ≥ 30	anti-dsDNA	0.021	[48]
Costenbader K.H. et al.	2008	Boston, USA	190 SLE	NR	Risk of SLE	NS	[49]
Bonakdar Z.S. et al.	2011	Iran	40 SLE^b^	25–39.9 vs. 12.5–24.9 vs. <12.5 nmol/l	anti-dsDNA	<0.001	[50]
Mok C.C. et al.	2012	China	290 SLE^c^	<30 vs. < 15	anti-dsDNA, anti-C1q	0.02 0.02	[51]
Thudi A. et al.	2008	Texas, USA	37 SLE	>47 nmol/l vs. <47 nmol/l	anti-dsDNA	0.0069	[52]
AlSaleem A. et al.	2015	Saudi Arabia	28 SLE children (26 female, 2 male)	51.1 ± 33.6	anti-dsDNA, ANA	NS NS	[53]
Muller K. et al.	1990	Denmark	35 Sjogren	28.2 ± 12.4	IgM RF, IgA RF	<0.05 NS	[54]
Hajas A. et al.	2015	Hungary	125 MCTD, 48 CTRL^d^	26.16 ± 13.5	anti-U1-RNP, anti-CL IgA	0.022 0.015	[55]
Zold E. et al.	2008	Hungary	161 UCTD	NR; hypovitaminosis D	anti-U1-RNP, anti-SSA, anti-CCP	0.024 0.029 0.0001	[56]
Paupitz J.A. et al.	2010	Brazil	23 APS^e^, 23 CTRL^f^	21.64 ± 11.26 vs. 28.59 ± 10.67 mg/dl	IgG anti-CL IgM anti-CL	0.222 0.535	[57]
Arnson Y. et al.	2011	Israel	327 SSc, 141 CTRL^g^	18.2 ± 13.2 12.7 ± 6.6 16.8 ± 12.3	ANA, Anti-SCL70, RF	NS NS <0.001	[58]

SLE = Systemic lupus erythematosus; ANA = Antinuclear antibodies; anti-dsDNA = Anti-double stranded DNA antibodies; APS = Antiphospholipid syndrome; PV = Pemphigus vulgaris; IgM RF = IgM rheumatoid factor; IgA RF = IgA rheumatoid factor; anti-CL IgA = anti-cardiolipin IgA; RNP = ribonucleoprotein; anti-SSA = anti-Sjögren's-syndrome-related antigen A; anti-CCP = Anti-cyclic citrullinated peptide antibody; SSc = Systemic sclerosis; anti-SCL70 = anti-topoisomerase I; RF = Rheumatoid factor

^a^Healthy matched controls; n=14 ANA-positive^^^, n=18 ANA-negative^^^^

^b^5 mild vitamin D deficiency, 25 moderate vitamin D deficiency, 7 severe vitamin D deficiency

^c^277 vitamin D insufficiency (<30 ng/ml), 77 vitamin D deficiency (<10 ng/ml)

^d^age- and sex-matched healthy controls

^e^pre-menopausal women

^f^age- and race-matched healthy controls

^g^healthy controls

^h^Correlation between vitamin D levels and positive CTD antibodies

### Inflammatory arthritides

In the Women’s Iowa Health Study, i.e., a prospective cohort of 29,368 women, it was demonstrated that vitamin D food intake was inversely associated with the risk to develop rheumatoid arthritis (RA) (*n* = 152) [59]. However, in smaller studies, no association was found. Firstly, in 722 patients with RA from the Nurses’ Health Study and Nurses’ Health Study II [49], and secondly, in the study by Nielen et al. (*n* = 79), no association between vitamin D deficiency and later development of RA was observed [60]. Feser et al. found in a healthy population at increased risk for RA that 25(OH)D levels were not associated with the presence of RA-related autoantibodies (76 subjects being positive for anti-CCP and/or RF) (*P* = 0.15) [61]. In contrast, RA patients with severe vitamin D deficiency (serum levels ≤15 nmol/L) (*n* = 15) were shown to test more frequently positive for RF (100%) when compared to RA patients with normal vitamin D serum levels (*n* = 200) (77.5%) (*p* = 0.05) [62]. In a 10-year prospective cohort study of 775 RA, 738 ankylosing spondylitis (SPA), and 721 psoriatic arthritis (PsA) patients, 25(OH)D levels, RF, and anti-cyclic citrullinated peptide (anti-CCP) antibodies were measured [63]. Levels were compared to 677 subjects without inflammatory rheumatic diseases. Five hundred twenty-eight RA patients (68.1%) were found to be RF positive and 482 RA patients (62.2%) anti-CCP positive. The prevalence of anti-CCP positivity was significantly higher in the 25(OH)D deficiency group (<20 ng/mL) (*p* < 0.022) compared to patients with sufficient levels of vitamin D [63]. Subclinical gut inflammation may be present in patients with SPA. In a cross-sectional study by Teichman et al., 47/76 patients with SPA tested positive for human anti-tissue-transglutaminase-IgA (htTG) antibodies. These patients had significant lower 25(OH)D levels compared to SPA patients that tested negative for anti-htTG antibodies (mean 17.4 vs. mean 41.5 ng/mL; *p* < 0.005) [64].

In conclusion, the relation between vitamin D food intake and the risk of development of RA later in life is controversial. Furthermore, some studies suggest that there is an inverse correlation between vitamin D levels and the presence of RA-related autoantibodies (RF and/or anti-CCP) in patients with RA (Table 6) and/or anti-TTG antibodies in SPA.

**Table 6:** Studies in which vitamin D levels are correlated to autoantibodies associated with inflammatory arthritides

Author	Year	Country	Subjects	Vitamin D (ng/ml)	Vitamin D effect / Autoantibodies	*P* value^d^	Reference
Merlino L.A. et al.	2004	USA	152 RA +, 29.368 RA -	NR	Risk of RA (RR 0.67)	0.05	[59]
Nielen N.M. et al.	2006	The Netherlands	79 RA, 79 CTRL^a^	<20 nmol/l	Risk of RA	NS	[60]
Costenbader K.H. et al.	2008	Boston, USA	722 RA	NR	Risk of RA	NS	[49]
Feser M. et al.	2009	USA	79 RA, 154 CTRL^b^	26.89 ± 10.04 vs. 25.30 ± 9.01	Anti-CCP and/or RF	0.15	[61]
Haga H.J. et al.	2013	Denmark	302 RA	≤15 nmol/l	RF	0.05^†^	[62]
Urruticoechea-Arana et al.	2015	Spain	775 RA, 738 SPA and 721 PsA, 677 CTRL^c^	<20 ng/ml	RF, Anti-CCP	0.022	[63]
Teichman J. et al.	2010	Germany	76 SPA, 116 PsA	17.4 ± 13.19 vs. 41.5 ± 44.21	htTG	<0.005	[64]

NR = not relevant; RR = Relative Risk; NR = Not reported; RA = Rheumatoid Arthritis; SLE = Systemic lupus erythematosus, anti-CCP = Anti-cyclic citrullinated peptide antibody; SPA = Ankylosing Spondylitis

^a^healthy blood donors matched for age, sex, and time of donation

^b^autoantibody-negative controls

^c^non-CIRD (chronic inflammatory rheumatic diseases) subjects

^d^Correlation between vitamin D levels and positive inflammatory arthritides antibodies

^e^compared vitamin D ≤15 nmol/l (n=15) to normal vitamin D levels (n=200)

### Multiple sclerosis

Low levels of vitamin D are common in patients with multiple sclerosis (MS) and are associated with more severe disability and an increased risk for relapses of the disease [65]. Also, the geographical distribution of MS suggests that people living in areas were vitamin D levels are low are more prone to develop MS. Furthermore, vitamin D supplementation may correct immunological abnormalities that are observed in MS [66, 67]. As a consequence, vitamin D supplementation is currently being studied for the prevention of disease relapses [68]. An association between low vitamin D levels and MS-related antibodies are, however, rarely reported. In a cross-sectional study of pediatric-onset MS, vitamin D status was found to be related to antibody levels to cytomegalovirus (CMV), while antibodies to other viruses (Epstein–Barr virus, herpes simplex virus 1 or 2) were not found to be associated with vitamin D levels [69]. Epstein–Barr virus (EBV) is postulated to play an important role in the pathogenesis of MS by infecting B cells [70]. Importantly, antibodies against the EBV antigens are associated with an increased risk of developing MS later in life [71]. Salzer et al. showed an inverse association between vitamin D levels and antibody titers against EBV in MS patients [72]. Décard et al. investigated serum 25(OH)D levels and EBV immunoreactivity in 25 individuals who donated blood prior to the first clinical MS manifestation [73]. Cross-sectional analyses revealed a gradual decrease of vitamin D levels and increase of anti-EBNA-1 IgG titers before the first clinical manifestation of MS. Furthermore, Disanto et al. showed decreased anti-EBNA-1 IgG levels after 12 weeks of vitamin D supplementation in 15 relapsing–remitting MS (RRMS) patients (*p* = 0.016) [74]. Another study by Najafipoor et al. examined the effect of vitamin D supplementation for 6 months on the anti-VCA IgG and anti-EBNA-1 IgG levels in 40 RRMS patients [75]. They found six patients (15%) with a decline in the level of anti-VCA titers and six patients (15%) with a reduction in anti-EBNA1 titers in the supplemented group. There was, however, no significant relationship between the vitamin D levels and antibody levels (VCA IgG *p* = 0.420; EBNA1 IgG *p* = 0.853).

In summary, both vitamin D levels and autoantibodies against EBV are reported to be important in the pathogenesis of MS. A causal relation between these two etiological factors is suggested in several studies (Table 7).

**Table 7:** Studies in which vitamin D levels are correlated to autoantibodies associated with Multiple sclerosis (MS)

Author	Year	Country	Subjects	Vitamin D (ng/ml)	Vitamin D effect / Autoantibodies	*P* value^d^	Reference
Mowry E.M. et al.	2011	USA	120 MS^a^/CIS, 20 CTRL	NR	anti-CMV, anti-EBV, HSV-1/-2	0.004 NS	[69]
Salzer J. et al.	2013	Sweden	192 MS, 384 CTRL	NR	anti-EBNA1	0.03	[72]
Décard B.F.et al.	2012	Germany	25 pre-CIS, 25 CTRL^b^	NR	anti-EBNA1	<0.01	[73]
Disanto G. et al.	2013	The Netherlands	15 RRMS^c^	NR	anti-EBNA1	0.016	[74]
Najafipoor A. et al.	2015	Iran	40 RRMS (27 vit. D suppl., 13 CTRL)	NR	anti-VCA IgG, anti-EBNA1	0.420 0.853	[75]

NR = Not reported; CIS = clinically isolated syndrome (prior to first clinical MS manifestation); CMV = cytomegalovirus; EMB = Epstein Barr Virus; HSV =

Herpes Simplex Virus; EBNA = Epstein-Barr Nuclear Antigen; RRMS = Relapsing remitting MS

^a^Pediatric-onset MS

^b^age- and gender-matched healthy blood donors

^c^vitamin D supplementation (n=27) and age- and gender-matched healthy blood donors

^d^Correlation between vitamin D levels and positive antibodies

### Other studies

Inflammatory bowel disease (IBD) is another disease that is linked to low vitamin D levels [17, 76]. A prospective study by Santos-Antunes et al. found an inverse relationship between vitamin D levels and ANA positivity in IBD patients [77]. The presence of ANA has also been shown to correlate with low serum vitamin D levels in patients with leprosy (*p* < 0.001) [78]. Ritterhouse et al. observed that ANA-positive healthy controls are significantly more likely to be deficient in vitamin D levels than ANA-negative healthy controls (*p* = 0.011) [46]. Nuti et al. found that 24 women with osteoporosis in the presence of coeliac disease and high levels of serum IgG anti-gliadin antibodies (IgG AGA) and tissue transglutaminase antibodies (anti-TTG) had lower serum 25(OH)D levels compared to 231 women with primary osteoporosis without coeliac disease (*p* < 0.01) [79]. They also found an inverse correlation between the serum 25(OH)D levels and the serum anti-TTG titers in these 24 women with primary osteoporosis and coeliac disease (*p* < 0.001). Karakan et al. investigated the presence of serum IgA anti-endomysial (IgA EMA) antibodies in relation to serum 25(OH)D levels in 135 patients with idiopathic osteoporosis and found that 13 patients (9.6%) which tested positive for IgA EMA had lower serum 25(OH)D levels compared to IgA EMA-negative patients (*p* < 0.01) [80]. However, none of these 13 patients had findings consistent with coeliac disease. Furthermore, in a cross-sectional study by Ota et al. of 133 women with recurrent pregnancy losses, a significantly higher prevalence of anti-phospholipid antibodies and TPO antibodies was found in 63 patients with low vitamin D levels (<30 ng/mL) compared to 70 patients with normal vitamin D levels (*p* < 0.05 each) [81]. A prospective study by Njemini et al. investigated the prevalence of several organ-specific and non-organ-specific autoantibodies and serum 25(OH)D levels in 152 unselected Cameroonians [82]. No association between the presence of autoantibodies and serum 25(OH)D levels could be found in this African population. Finally, Carvalho et al. observed anti-vitamin D antibodies in a subset of patients with several different autoimmune diseases [83], but could not find a significant difference in serum 25(OH)D levels between the anti-vitamin D antibody-positive (*n* = 7) and anti-vitamin D-negative (*n* = 164) patients.

In conclusion, an inverse correlation between serum vitamin D levels and the presence of autoantibodies in several other conditions has been suggested as well (Table 8).

**Table 8:** Studies in which vitamin D levels are correlated to antibodies in other autoimmune diseases

Author	Year	Country	Subjects	Vitamin D (ng/ml)	Autoantibodies (AAb)	*P* value^d^	Reference
Santos-Antunos J. et al.	2016	USA	68 IBD (56 CD, 12 UC)	<4 ng/ml	ANA	<0.05	[77]
Ribeiro S.L.E. et al.	2012	Brazil	87 Leprosy (22 ANA +, 55 ANA -)	31.20 ± 10.8 vs. 53.03 ± 24.99	ANA	<0.001	[78]
Nuti R. et al.	2001	Italy	53^a^ IgG AGA + (24 TG-Ab +, 29 TG-Ab -)	17.8 ± 7.2 vs. 55.1 ± 20.3 nmol/l	IgG AGA, Anti-TG	<0.01	[79]
Karakan T. et al.	2007	Turkey	135 low BMD^b^ (13 EMA +, 122 EMA -)	11.6 ± 1.89 vs.	IgA EMA	<0.01	[80]
Ota K. et al.	2014	USA	133 RPL (63 vit D. def., 70 normal vit. D)	19.8 ± 4.88 <30 ng/ml vs.	APA^c^, ANA, anti- ssDNA, anti-TPO	<0.05	[81]
Njemini R. et al.	2002	Belgium	152 Cameroonians	≥30 ng/ml 12.5 ± 3.2 vs.	Various AAb^e^	NS	[82]
Carvalho J.F. et al.	2007	Israel	171 SLE, 56 APS, 18 PV, 94 CTRL^f^	21.1 ± 7.7 mg/l 28.4 ± 9.6 vs. 26.4 ± 13.9	anti-vitamin D Ab,	NS	[83]

IBD = Inflammamtory Bowel Disease; CD = Crohn’s disease; UC = Ulcerative colitis; BMD = Bone mineral density; IgA EMA = IgA anti-endomysial; RPL = Recurrent pregnancy losses; APS = Antiphospholipid syndrome; APA = Antiphospholipid antibody

^a^Postmenopausal osteoporotic women

^b^Idiopatic

^c^Any IgG or IgM antibodies to phospholipids

^d^Correlation between vitamin D levels and positive antibodies

^e^Organ-specific and non-organ-specific autoantibodies

^f^Comparing n=7 anti-vitamin D positive and n=164 anti-vitamin D negative

## Discussion

The aim of our study was to investigate the relation between vitamin D levels in patients with ASIA in relation to silicone implant incompatibility and the presence of autoantibodies. Vitamin D deficiency or insufficiency was found to be related to the presence of autoantibodies in our patients with silicone breast implants. A critical limitation to this finding is that we did not perform a study in control patients. Whether vitamin D deficiency is also related to the presence of autoantibodies in healthy control women remains therefore unknown. Furthermore, we reviewed the literature regarding an association between vitamin D deficiency and the development of autoantibodies in several other (autoimmune) diseases. To our knowledge, this link has not been reviewed before. Conflicting data regarding the relation between vitamin D levels and autoantibodies were found since low vitamin D levels were linked to the presence of autoantibodies and/or the antibody titers in some, but not all studies. Thirty-one studies described a significant negative correlation between serum vitamin D levels and autoantibodies: ten for anti-thyroid antibody levels [31–40], nine for autoantibodies in connective tissue diseases [46, 48, 50–52, 54–56, 58], two for RA-related autoantibodies (RF and/or anti-CCP) [62, 63], one for anti-TTG antibodies [64], four for autoantibodies against EBV in MS patients [69, 72–74], and five for autoantibodies in several other (autoimmune) diseases [77–81]. Thirteen studies could, however, not confirm this correlation [41–45, 49, 53, 57, 60–61, 75, 82–83].

Vitamin D deficiency has been underestimated for many years as a health problem. Importantly, the role of vitamin D insufficiency and/or deficiency in the development of different autoimmune diseases is at present increasingly studied [47, 84]. It has been demonstrated that many cells of the immune system express vitamin D receptors (VDRs), e.g., monocytes and dendritic cells (DC) [18]. Whereas resting T and B cells express negligible VDR, activation of these cells results in upregulation of VDR expression [19]. Furthermore, activated B cells express CYP27B1 and CYP24A1 enzymes, which are responsible for the synthesis and breakdown of the active metabolite of vitamin D, thereby regulating vitamin D activity in the microenvironment [19, 68, 85]. Importantly, Chen et al. demonstrated that 1,25(OH)_2_D has potent direct effects on B cell responses, inhibiting proliferation, generation of class-switched memory B cells, plasma cell differentiation, and Ig production [19]. Furthermore, they demonstrated that 1,25(OH)_2_D induced increased apoptosis of proliferating B cells [19]. Chen et al. therefore suggested that vitamin D deficiency could contribute to B cell hyperactivity, breakdown of B cell tolerance, and production of autoantibodies in SLE patients [19]. Subsequently, it was demonstrated in in vitro experiments that synthesis of IgM, IgG, and IgA by various stimuli was inhibited in the presence of 1,25(OH)_2_D [19]. These in vitro inhibitory effects of vitamin D are, however, not confirmed in vivo [86–88]. Ritterhouse et al. found that SLE patients with more pronounced B cell activation had lower mean 25(OH)D levels compared to SLE patients with less B cell activation [46]. Importantly, they also found that patients with vitamin D deficiency had higher mean serum IFNα activity than patients without a vitamin D deficiency [46]. IFNα is produced in large amounts by activated dendritic cells (DCs) and has multiple pro-inflammatory effects on autoreactive cells and is therefore considered to be a central player in the progression to and maintenance of autoimmunity [89]. Excessive dendritic cell activity could therefore be another hypothesis for the production of autoantibodies in vitamin D-deficient patients. Whether these pathophysiological hypotheses are relevant for the development of autoimmune diseases is, however, at present not clear.

## Conclusion

Vitamin D may act as a regulatory agent of the immune system. Vitamin D deficiency is found to be related to the presence of autoantibodies in patients with silicone implant incompatibility syndrome. In line with our study, we found several reports that suggested that there is evidence that vitamin D levels are related to the presence and/or levels of autoantibodies in thyroid autoimmune diseases (AITDs), connective tissue diseases (CTDs), multiple sclerosis (MS), and several other (autoimmune) diseases. Randomized, controlled trials must be performed to elucidate whether vitamin D supplementation is beneficial as preventive therapy in patients with silicone breast implants to inhibit the development of autoantibodies.
